# Promoting Evidence-Based Practice for Improved Occupational Safety and Health at Workplaces in Sweden. Report on a Practice-Based Research Network Approach

**DOI:** 10.3390/ijerph17155283

**Published:** 2020-07-22

**Authors:** Irene B. Jensen, Elisabeth Björk Brämberg, Charlotte Wåhlin, Christina Björklund, Ulric Hermansson, Malin Lohela Karlson, Liselotte Schäfer Elinder, Peter Munck af Rosenschöld, Tarja Nevala, Ned Carter, Bodil Mellblom, Lydia Kwak

**Affiliations:** 1 Unit of Intervention and Implementation Research for Worker Health, Institute of Environmental Medicine, Karolinska Institutet, 171 77 Stockholm, Sweden; elisabeth.bjork.bramberg@ki.se (E.B.B.); Charlotte.Wahlin@regionostergotland.se (C.W.); christina.bjorklund@ki.se (C.B.); lydia.kwak@ki.se (L.K.); 2 Department of Public Health and Community Medicine, Sahlgrenska Academy, University of Gothenburg, 405 30 Gothenburg, Sweden; 3 Occupational and Environmental Medicine Center and Department of Health Medicine and Caring Sciences, Unit of Clinical Medicine, Linköping University, 581 83 Linköping, Sweden; 4 Department of Clinical Neuroscience, Centre for Psychiatry Stockholm Center for Dependency Disorders, Stockholm, Sweden Department of Public Health, Karolinska Institutet, 171 77 Stockholm, Sweden; ulric.hermansson@ki.se; 5 Department of Environmental and Occupational Medicine, Uppsala University and Region Vastmanland—Uppsala University, Centre for Clinical Research, Hospital of Vastmanland Vasteras, 721 89 Vasteras, Sweden; malin.lohela.karlsson@regionvastmanland.se; 6 Department of Global Public Health, Karolinska Institutet and Centre for Epidemiology and Community Medicine, 171 77 Stockholm, Sweden; liselotte.schafer-elinder@ki.se; 7 Swedish Association of Occupational Health and Safety, 102 04 Stockholm, Sweden; peter.munck@foretagshalsor.se; 8 Swedish Agency for Government Employers, 103 33 Stockholm, Sweden; Tarja.Nevala@arbetsgivarverket.se; 9 Swedish Association of Local Authorities and Regions, 118 82 Stockholm, Sweden; nedcarter52@outlook.com; 10 The Confederation of Swedish Enterprise, 114 82 Stockholm, Sweden; Bodil.Mellblom@svensktnaringsliv.se

**Keywords:** occupational health services, practice-based research network, guidelines, workplace interventions, occupational safety and health, implementation research

## Abstract

Despite the rapid growth in research and R&D expenditures, the translation of research into practice is limited. One approach to increase the translation and utilization of research is practice based research networks. With the aim of strengthening evidence-based practice (EBP) within occupational health services in Sweden (OH-Services), a practice-based research network (PBRN-OSH) was developed. The PBRN-OSH includes researchers and representatives from end-users. This paper reports on the development, outputs and lessons learned in the PBRN-OSH. The PBRN-OSH resulted in several practice-based research projects as well as different measures to ensure EBP in OSH such as the governmentally sanctioned national guidelines for the OH-services. Moreover, results show that the competence in EBP increased among practitioners at the OH-services. Conducting research in a PBRN is more resource demanding; however, this does not imply that it is less cost effective. To succeed in increasing the utility of research findings via PBRN, resources must be invested into an infrastructure that supports collaboration in the PBRN, including costs for a variety of means of dissemination. Further, translation activities need to be included in academic career paths and reward systems if a major improvement in the impact and return of investments from research is to be expected.

## 1. Introduction

### 1.1. Research into Practice—Translation and Dissemination

Sweden is among the leading countries when it comes to Research and Development (R&D) expenditures. Spending is well above the EU average, with expenditures of about 1% of Gross Domestic Product (GDP) in the public sector and 2% of GDP in the private sector [1]. A rapid growth in the number of researchers and research publications can also be seen internationally. However, an increase in scientific production does not necessarily imply a greater impact on society, as the translation of scientific findings into practice is a painstaking endeavour [2]. This is not only due to the time-consuming process of getting a scientific paper published and cited (i.e., dissemination in the scientific community) but also the long process of communicating findings and translating them into practice [3]. Studies have shown that it can take up to seventeen years for research findings to reach end users. In the worst cases, potentially effective interventions are never implemented [4].

The utility and on return of investments, as well as the quality and international impact of the research produced, are important when evaluating the societal benefit of research investments [1]. As accentuated by the EU as well as the Swedish government, the overall low utilisation of research results is worrying. Given the rapid growth in research and high R&D expenditures, there is a need to understand what can be done to improve the translation of scientific knowledge into practice.

The 2004 NIH initiative for a new “road map” in medical research emphasized the importance of translating research findings into practice. It describes research studies in-between pure clinical studies and clinical practice, the so-called “Blue Highways” [3]. The latter are defined as practice-based research applying controlled intervention studies where the intervention is being carried out and administered in cooperation with end-users. They further involve dissemination and implementation research together with systematic reviews, meta-analysis, and guideline development. Including the end-user (e.g., the practitioners at the clinics) in the research is a promising strategy for bridging this gap. Growing interest in closing the gap between research and practice has brought attention to the benefits of participatory approaches for the dissemination and implementation of research findings [5].

Participatory research, also called practice-based research (PBR), is applied in many contexts such as communities, workplaces, and health care. PBR is commonly organised in the form of practice-based research networks (PBRN). The research carried out in these networks is often multidisciplinary and conducted (i) in close collaboration with relevant partners outside the research community, (ii) in context (in health care clinics, hospitals, workplaces, etc.), and (iii) with social accountability, ensuring relevance for the end-user. End-users interests are thus integrated into all steps of the research process. The major strength of a PBRN is that the experiences of end-users are incorporated when formulating relevant research questions and designing studies that are applicable to everyday practice. Involving end-users in research has been shown to be an important strategy for successful implementation and utilisation of research findings [6].

### 1.2. The Importance of a Practice-Based Research Network in Occupational Safety and Health

One area where there is a great need of increasing the utilisation of research findings into practice is occupational safety and health. Decades of research have shown the significance of good working conditions for sustainable employee health, and evidence suggests that workplace interventions are good investments for promoting employee work ability and preventing work-related disability. However, just as in health care, the utilisation of research results for promoting occupational safety and health (OSH) is low [7]. An EU report highlights that, in order for research to have an impact on workers’ safety and health, there is a great need to improve the translation of OSH research findings into practice, both in the occupational health services (OH-Service) and at workplaces [7].

There are great challenges facing the translation of OSH-research findings into practice. These include a scarcity of evidence-based workplace interventions and the complexity of OSH interventions, which often span multiple organisational levels. The wide variety of settings, which include many sectors, workplaces, and professions, makes the transfer of methods from one setting to another even more challenging. In addition, contextual factors influencing implementation, such as society, organisations, economic conditions, laws, and regulations, are continuously changing. A PBRN could be an effective solution to these problems and could ultimately increase the utilisation of research findings and facilitate evidence-based practice in OSH [6,8]. With a PBRN, it is possible to improve the planning and execution of studies that are relevant to users [8,9,10,11]. Cooperating in a PBRN (i) provides easier access to the “arenas” where research can be conducted, (ii) increases the likelihood that interventions are feasible and applicable to the reality of the workplace, (iii) promotes knowledge exchange between researchers and users, (iv) speeds up dissemination and utilisation of research findings, and (v) increases the commitment of users (managers/employees) to the study in progress [8,9,10,11].

### 1.3. Occupational Health Services

A key partner in a PBRN which aims to facilitate evidence-based practice in OSH is the occupational health service (OH-service). In Sweden, the OH-service is an expert resource helping employers and employees to achieve a sustainable and healthy working life. The OH-service is defined in the Swedish Work Environment Act section 2c, as an independent expertise resource for OSH and vocational rehabilitation [12].

OH-services in Sweden are either external private companies or in-house, with the clinical staff being employed by the company they work for. It is not mandatory for employers to have an OH-service, but it is mandatory to have access to expertise in the management of OSH at the workplace. OH-services are members of the branch association for Swedish occupational health services, The Swedish association of Occupational Health and Safety [13]. The association is responsible for the accreditation of OH-services. About 65% of all employees in Sweden have access to an OH-service. Access to OH-services is universal for public sector employees in Sweden. The majority of those with no access are employed in small enterprises (≤50 employees). Swedish OH-services employ about 4000 professionals in a range of professions with specialist competence in OSH. The professions that work in the OH-services are specialized in OSH and have a unique knowledge of workplaces and job designs in different sectors.

## 2. The PBRN-OSH Program

To bridge the gap between research and practice in OSH, we started the PBRN-OSH program in 2011. We obtained dedicated funding for building up the program over six years from two independent funding agencies in Sweden: AFA-Insurance, which is an insurance company owned by labour market parties [14] and the Swedish Research Council for Health, Working Life and Welfare [15]. The mission of the program was to develop and promote the use of evidence-based measures in the everyday work of OH-services in supporting OSH at workplaces. To achieve this, we developed a knowledge synthesis and translation system and a prevention support system using the Interactive Systems Framework for dissemination and implementation (ISF) as a guiding framework [16].

**Figure 1:**
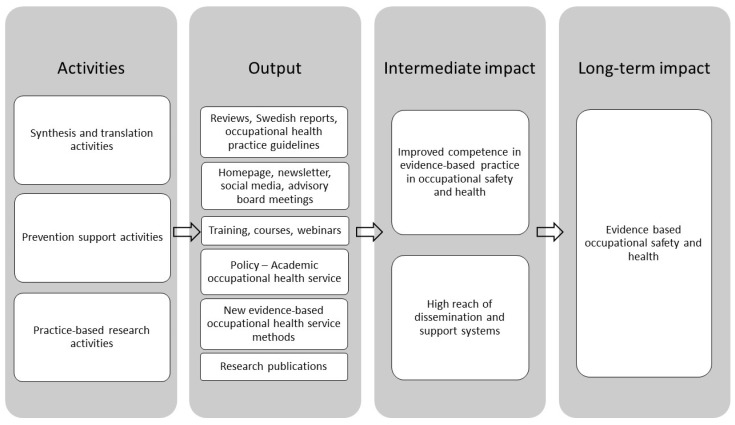
Logic model of the practice-based research network (PBRN-OSH) program.

AIM: The aim of this paper is to describe the development, outputs, and lessons learned from the PBRN-OSH program during the first six years. The paper also describes the impact of the program on translating OSH-research into practice. In Figure 1, the postulated mechanism of change is depicted in a logic model using the Interactive Systems Framework (ISF) as a guiding framework. The ISF includes three systems that support dissemination and implementation of evidence-based interventions, methods, and policies [16] described in more detail in Section 2.2.

### 2.1. The Partners in the PBRN-OSH Program

**Figure 2:**
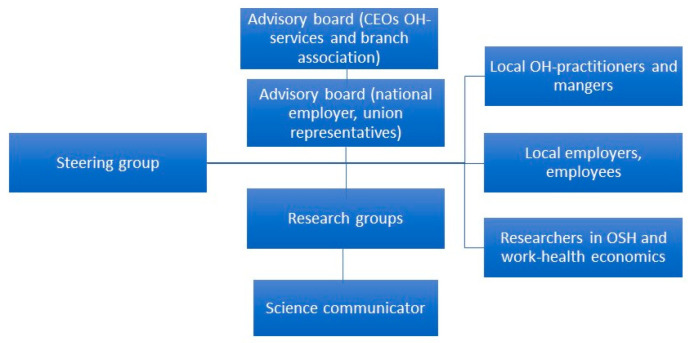
Organisation of the PBRN-OSH.

The network is led by a steering group of 6‒8 researchers with different areas of expertise in OSH from three research departments and institutes (Figure 2). The network further has two advisory boards consisting of stakeholders not working in academia. One of the advisory boards consists of OH-service representatives, mainly from higher management, and the the CEO of the Swedish association of Occupational Health and Safety. The other advisory board consists of representatives of Swedish national employer organisations covering the private sector, public and state sectors, and union representatives. The advisory boards function as channels for knowledge exchange between researchers and practitioners. These contacts facilitate participation in research studies and the dissemination and communication of research findings.

Separate meetings between the steering group and each of the advisory boards take place every six months. The purpose of the meetings is, firstly, to discuss current challenges in OSH, including what knowledge gaps between research and practice need to be addressed and secondly, to present new research findings to the partners. During these meetings, partners and researchers formulate tentative research questions and discuss possible studies. In addition to the researchers in the steering group, the PBRN-OSH involves additional researchers who are involved in the program in a variety of ways, and a communicator.

### 2.2. The Structure of the PBRN-OSH Program

The rationale for using the Interactive Systems Framework (ISF) as a guiding framework in the development of the PBRN-OSH program is that it describes the contributions that researchers, stakeholders, practitioners, and others can make to bring evidence-based methods into practice. The ISF includes three systems that support dissemination and implementation of evidence-based interventions, methods, and policies [16]. The Prevention Synthesis and Translation System (PSTS) distils information generated by research and prepares it for dissemination and implementation by end users. The Prevention Support System (PSS) supports the work of those who will put the innovations into practice, for example through training and technical assistance. The Prevention Delivery System (PDS) implements innovations into practice. The activities and outputs of these systems are described below.

#### 2.2.1. Prevention Synthesis and Translation System

A PSTS was developed with the aim of summarizing scientific knowledge regarding evidence-based methods in OSH and translating this knowledge into popular science publications and practice-based guidelines for the OH-services.

The output of the PSTS system up to 2019 was 31 popular science publications in Swedish, seven systematic reviews and 19 research reports, four books, and one patient booklet. The systematic reviews were translated into five occupational health practice guidelines and three work-health economic analysis tools (see Table 1). In addition, short information leaflets were produced for all guidelines to provide employers with an overview of the recommendations given in the guideline. A process evaluation of the model for developing guidelines was done during the whole development process of the first guideline. The process evaluation is published in full in Kwak L. et al. (2017) [17]. In brief, the evaluation showed that the development process is a feasible model to develop guidelines by including end-users in the process. The model was accordingly applied to the development of additional guidelines within the field of OSH.

The intermediate impact of the developed guidelines was assessed by evaluating the dissemination of the first guideline (Guideline for non-specific low back-pain at the workplace) ten months after its release [18]. A questionnaire was sent to a random sample of occupational health practitioners (*n* = 467) from 71 different OH-services. The questionnaire contained statements regarding practitioners’ perceptions of the guideline and statements regarding barriers that hinder implementation of the guideline. Respondents were asked to what extent they agreed with the statements. Of the 339 (72.6%) respondents who replied to the questionnaire, 45% indicated that they were aware of the guideline. Of these, 78.2% reported that they fully or partly adhered to the guideline (Table 2). Most of the respondents had positive perceptions of the guideline. The main perceived barrier was that working according to the guideline was time-consuming and that it was difficult to change routines in everyday practice.

#### 2.2.2. Prevention Support System

Practitioner level: A PSS was developed with the aim of providing support to OH-practitioners and employers. The PSS used training courses and webinars to build competence to apply EBP in everyday work.

Organisational level: The PSS also aimed to increase an organisation’s incentive to work according to evidence-based practice. This was done by establishing Academic OH-services, designed to support and secure evidence-based practice in OH-services. It also aimed to create a channel to improve contact between the OH-services and the PBRN-OSH network. One way of creating a structure/organisation for the successful implementation of evidence-based methods is by providing certification [6]. Accordingly, in cooperation with the branch association for Swedish OH-services, we formulated a set of criteria for how to become a certified academic OH-service. OH-services needed to fulfil the following criteria: (i) they should work evidence-based, for example, in accordance with the occupational health practice guidelines; (ii) they should collaborate with researchers in research studies, and (iii) they should offer employees opportunities for skills development. The recruitment and certification processes were carried out in collaboration with the Swedish business association of OH-services.

Science communication: As previous studies have reported, social media have become a platform for science communication, for learning and networking [19]. To provide information in the best possible way and facilitate support to practitioner, a communication expert was appointed, and a communication plan was developed. This plan included several information strategies and channels, including social media.

The output of the PSS is conceptualized as the number of training courses, webinars, and communication platforms developed and used in the program (see Table 3). One course aimed at general capacity building to promote EBP. Other training courses aimed at innovation-specific capacity building related to the methods recommended in the guidelines. One example is the course in problem-solving dialogue that is recommended to facilitate the return-to-work of individuals on sick-leave due to common mental disorders. Courses were given between 1 and 34 times during the six years of the program, with the number of participants ranging from 15‒900. One to two launching seminars were given for each occupational health practice guideline for innovation-specific capacity building. Each seminar was filmed and developed into a webinar which can be downloaded from the program’s webpage and used for online learning by, for example, OH-practitioners, employers, and students. Researchers involved in the program must also regularly give presentations on evidence-based practice in OSH at conferences and seminars targeting practitioners.

The output of the PSS also included the development of a communication platform. The platform included a webpage, newsletter, blog, and social media. A Swedish webpage [20] to support EBP in OH-service by disseminating new research, evidence-based tools and measures, and information about training courses was launched in 2014. It targets a variety of stakeholders such as OH-practitioners, OH-managers, employers, managers, and human relation officers. It gives free access to webinars in the field and a QA-service by which visitors can send questions via e-mail about OSH related topics and receive answers from experts. An electronic newsletter targeting practitioners in OH-services and employers was created and started in 2014. The aim of the newsletter is to provide brief information in the field of OSH, for example, news related to ongoing research projects (including participant recruitment advertisements), research findings, and news about courses. The newsletter includes electronic links for further reading. It is distributed 4–5 times per year. In addition to the newsletter, a Swedish OSH-blog was launched in 2011 [21]. The purpose of the blog is to share thoughts about OSH, disseminate knowledge, and promote learning opportunities and forums for collaboration. Existing traditional channels provided by Karolinska Institutet were also used to disseminate information.

One important consequence (output) of the program is the partners’ involvement in disseminating information. Through the various partner organisations and their related media (press, homepages, newsletters, etc.).

### 2.3. Practice-Based Research Studies

**Figure 3:**
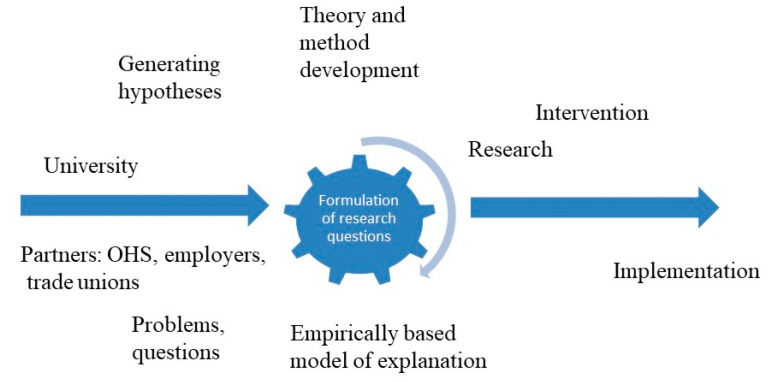
The PBRN-OSH research process.

The research process in PBRN-OSH is outlined in Figure 3, illustrating the interaction throughout the process.

When a research question is formulated and a decision made to design a study, the research group reaches out to local employers and OH-services and invites them to participate in a research study early in the process to ensure relevant input from the user perspective. When acceptance of the study is agreed between the parties, commitments are made by each stakeholder. In meetings with local partners, clear rules of practice-based research are set and agreed upon, as presented in Table 4. Agreements are made on when and where and how the interventions should be delivered, which items to include in questionnaires, time points for different deliverables from the study, etc. When funding has been obtained, the design of the study is finalized and pretested in real life settings (OH-service, workplace) in cooperation with the partners and the targeted setting. In order to ensure engagement and adherence to the agreement and study protocol at the setting, the study can be designed so that some outputs (not related to primary outcome) can be regularly fed-back to the involved organisations and study participants during the whole study period. Researchers work actively to maintain contact with the project leaders at the settings throughout the study. Results are always presented and discussed first with the organisations and then disseminated to study participants. In the intersection between researchers and partners, one may be challenged by the fact that partners urge for innovations or fixes of problems regardless of generating new knowledge for the good of the general. The importance of a written agreement before anything has started cannot be overemphasized.

A list of research studies conducted during the first six years of the program, in accordance with the PBRN-model, is provided in Supplementary File.

The majority of our internationally published research reports were summarised in Swedish, in a short communication series *Metoder för företagshälsa* (In English: *Methods for occupational health services*).

## 3. Impact of the PBRN-OSH-Program

### 3.1. Improved Competence in Evidence-Based Practice in OH-Services

At the start of the PBRN-OSH program (2011), we conducted a survey based on a random sample of OH-service professionals. The aim of the survey was to investigate the competence of working in accordance with evidence-based practice. The results demonstrated that in general, there is a low degree of collaboration between Swedish OH-services and academic institutions and a great need for improvement and training in EBP [22]. The purpose of the guideline for OH-services developed in the program was mainly to facilitate and thus increase the use of EBP in OH-services in Sweden. In the follow-up of the implementation of the guidelines, some problems hindering the use of the guidelines were reported by the practitioners (Table 2). A follow up three years later (2014) was conducted with the primary aim to investigate whether the development of evidence-based practice (EBP) in the Swedish OH-services had resulted in any changes in OH-professionals’ attitudes and knowledge towards EBP and in the EBP since the start of the PBRN-OSH program. Results revealed that OH-practitioners knowledge of EBP had increased and, further, that the organisational support, i.e., management facilitating the implementation of EBP, had decreased [23]. This was confirmed in the interviews with OH-service managers, who maintained that EBP was the responsibility of individual practitioners. Individual practitioners’ interest in EBP was viewed by the management, as the driving force for its implementation in clinical work. Notwithstanding, OHS managers considered that guidelines and collaboration with research units facilitates the use of EBP in OH-services. Overall, OH-service management and practitioners were positive towards EBP. However, the results indicated that important cornerstones for the successful development of EBP were missing. The lack of organisational support and resources (time, training, etc.) are serious barriers to implementing innovations [6]. Our findings underscore the need to support managers in how to successfully implement EBP in the organization.

### 3.2. Accreditation in EBP for OH-Services

To become a full member in the Swedish association for OSH (accredited OH-service), the service must fulfil a number of quality criteria [13]. After the first guideline for OH-services had been introduced, the PBRN-OSH network reached an agreement with the association that, to achieve accreditation, OH-services must work in accordance with EBP and apply the developed guidelines in their clinical practice. The opportunity to be certified as an academic OH-service was, as described earlier, introduced in 2015. However, for logistic reasons (time, cost coverage, and resources) the PBRN-OSH program could not continue with the certification process. As a result, only three OH-services have hitherto been certified.

### 3.3. National Guidelines for OH-Services

After developing and evaluating our model for producing guidelines for OH-services, we sought for this to be continued, preferably as a regular state activity, as the national guidelines for health care produced by the Swedish National Board of Health and Welfare. Several years later, after joint efforts of the partners within in the PBRN-OSH to promote the use of the guidelines with in the OH-services and to employers and meetings with representatives at the Ministry of employment and Ministry of social affairs, the guideline model became a governmental initiative to secure the continuation of evidence-based national guidelines for the Swedish OH-services. It has since 2018 been transferred to and implemented into the Swedish Agency for Work Environment Expertise (SAWEE) [24] regular activities, with support from our research group. In 2018‒2019, we produced a structured manual for the agency on how to develop guidelines in accordance with our evaluated model; existing guidelines were incorporated into the agency’s communication profile. This year (2020) the first two guidelines under the umbrella of SAWEE will be produced.

### 3.4. Increasing Evidence

The systematic reviews conducted have assisted us in focussing our research studies on areas where more research is needed before any gaps can be closed, e.g., interventions for preventing CMD at work. Moreover, several of our studies (e.g., interventions for back pain and CMD at work) have been included in several recent independent international systematic reviews contributing to the evidence in these areas.

## 4. Discussion and Lessons Learned

There are many challenges in the translation of research into practice. During the program, we have developed several important measures to promote the translation of OSH research into practice for target groups such as employers, workers, unions, and OH-services in Sweden. The practice-based research network (PBRN-OSH) developed in the program has given rise to several research projects as well as several measures to promote EBP in OH-services. These include national guidelines and the inclusion of EBP as a mandatory criterion in the accreditation of OH-services. Importantly, our results in the follow up of knowledge about and use of EBP in OH-services found that practitioners’ knowledge of EBP had increased [23].

Several lessons arise from our experience of developing the PBRN-OSH program and moving into more practice-based research. One lesson is the importance of ensuring sustainability. As with the present program, funding may be limited in time (six years for PBRN-OSH), which may seriously challenge the continuation of measures which have proved effective. Thus, it is important to have a plan to secure infrastructure support and continued financing and administration of measures. In our case, the continuing development and use of national guidelines for OSH has been ensured by our model being adopted by a government authority (SAWEE, [24]) devoted to disseminating EBP in OSH and OH-services. This arrangement was reached after several years of networking and lobbying, targeting stakeholders from labour market parties, ministries, and other government agencies. As researchers, we are not used to this type of work, but in our experience, the case for the successful dissemination of research has to be put forward by experts with authority in the field—if not, it is difficult to gain the attention of decision-makers [25]. A strategic decision was taken by the research group to devote part of the funding to communicating research findings to practitioners by building communication platforms (newsletter, homepage, etc.) In our experience, having a communication plan is a prerequisite for effectively communicating research, using appropriate media to reach different OSH target groups. As reported by previous studies, we found that social media have become an important platform for learning [19,26].

Research in PBRN poses challenges with regard to the role of the researcher and to research design [27,28]. As implementation science advances, it is questioned and debated on whether randomised controlled design is optimal in this type of studies, as well as its applicability in real world settings. It can however be difficult to get acceptance from the research community for a non-randomised study design in health intervention research such as OSH-research, with randomised controlled studies being the golden standard. The main themes of the debate concern the generalizability of the new knowledge produced by the research and the question of external and/or internal validity. In our experience, it is almost impossible to obtain the consent of workplaces to engage in studies using random assignment and/or control groups that do not receive any intervention. Similarly, study designs where, for example, some parts of the workplace have to wait for a long time before any intervention can take place (waiting list control design) are met with scepticism from partners. However, in the last decade, acceptance of non-randomised trials has increased in the field of dissemination and implementation research [27].

Another challenge related to scientific quality and evidence is that the interventions and assessments planned in studies must be adapted to and matched with the workplace’s internal setting and need to continue with business as usual to reach their production goals. That is why it is essential to have partner engagement from start to finish in the research process as depicted in Figure 3 above. The partners should not merely be seen as assisting parties in the dissemination and implementation of research. The bidirectional partnership required in a successful PBRN, i.e., that researchers and partners are equals, is initially challenging for both parties [29]. However, if time is devoted to learning how to communicate and consider each other’s viewpoints and skills, the joint goal of improving the targeted area (in our case OSH), can be reached. As highlighted in Gordan et al.’s review of PBRN(2019), new researchers entering a PBRN need to be mentored during their integration into the network [28]. In the PBRN-OSH program, we developed a PhD course in implementation science and wrote a textbook to facilitate the training in the translation of research into practice of young researchers. The research challenges posed by PBRN are, in turn, also a strength since the adaptions lead to higher involvement, acceptance, and adherence.

Our experience shows that one of the major challenges in PBRN studies is that the practitioners who are supposed to perform the intervention have difficulty with adherence, due to lack of time and resources and often also capacity/competence. To run a successful study in a workplace setting, the organisation and senior management must support the project fully. Equally importantly, they must have the skills and competence to properly facilitate and support the interventions tested in a research study. Management must provide the organisational conditions and resources for the practitioners to implement what is agreed on in the PBRN. Our experience indicates that lack of fidelity to the intervention is often caused by lack of support from the management, time constraints, and lack of competence. This is in line with the findings of previous research in the field of implementation science [30]. Similarly, practitioners must understand and meet the researchers’ needs regarding such factors as quality, the production of scientifically valid studies that can be published in scientific journals, time pressure, and tough funding requirements.

During the course of our work in building the PBRN-OSH, we have identified a number of basic conditions that must be met if partners are to be motivated. (1) The research must be seen as highly relevant to partners; (2) the researcher must take the time to learn about the setting and the everyday circumstances of the end-users (in this case workplace, OH-services), what they produce, and the conditions in which the employees work; (3) the study must be designed so that short term results and input to the workplace can be fed back at short and regular time intervals; (4) the feedback and insights gained from partners about their workplace must be respected and integrated into the study design; and (5) there must be a written contract setting out the agreements and obligations agreed on.

When working in a PBRN one sometimes encounters divergency in how innovation vs research is valued. Partners seek innovations helping them improve business or solve problems while researcher seek to explore and generate new knowledge. However, both concepts are needed and should be in action when conducting research in PBRN, especially in implementation research where the concepts are overlapping because there you seek to research how to effectively implement innovations into everyday practice. Primary care research and other community-based health care dominate in PBRN. However, the experience from the latter has been of great help in developing the PBRN-OSH, targeting practitioners in the Swedish OH-services and their associated workplaces. As far as we know, our network is the first in an OSH setting to be described and published. In line with previous studies, our work supports the contention that PBRN is beneficial from several perspectives [28,31]. It can result in greater relevance at societal level because studies are more likely to directly address current social problems. At user level (practitioners, businesses), it promotes increased cooperation and understanding of the importance of EBP and thereby facilitates the use of effective measures for OSH at workplaces. From a scientific perspective, it helps the researcher to identify and prioritise relevant research questions. It also raises the scientific quality of studies as a result of higher response rates to questionnaires, better adherence to the method, and by being tested on site in everyday business. This promotes acceptance from stakeholders and thereby facilitates dissemination and implementation at workplaces. As our results (and previous studies) show, working in PBRN also leads to increased utilization, which is an important factor in occupational health research [3].

**Figure 4:**
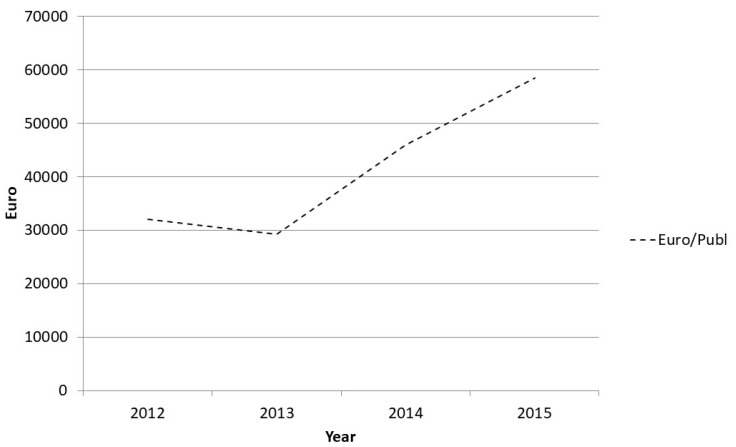
Cost per publications per full time researcher during the development of the program.

However, some challenges remain. Compared to traditional research conducted in a relatively closed academic research setting, PBRN requires researchers to devote time to communicating, networking, and meeting practitioners. This is time that would otherwise have been spent on academic research activities such as writing grant proposals, international research reports, etc. A cost follow-up of the program also shows that PBRN is costlier than traditional academic research. We have been continuously monitoring research productivity at the research unit for a number of years, measuring publications (the most commonly assessed research production output) against turnover in SEK. The results show clearly that PBRN increases costs. Costs per publication doubled in the period from the start of the program until 2015 (Figure 4).

**Figure 5:**
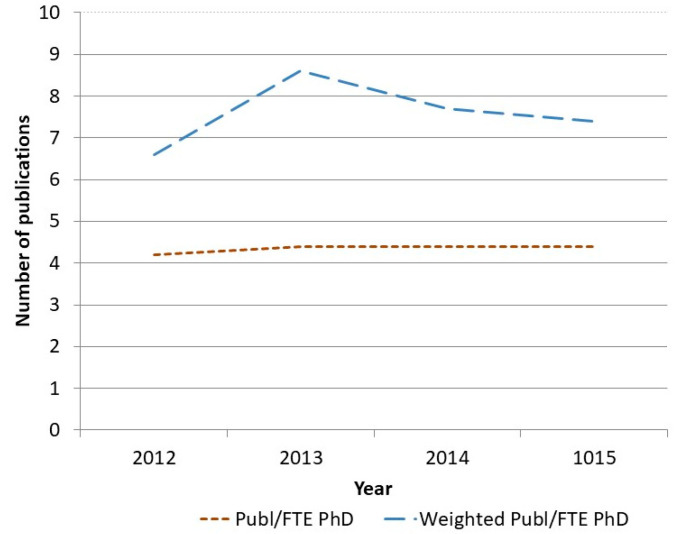
Mean number of publications per full time (FTE) researcher unweighted and weighted per type of publications. Weighted scores: Original international publication score 2, popular science reports in Swedish score 1, systematic reviews and guidelines in Swedish score 1.5. FTE = full time employed.

Why is this the case? As described in the above-mentioned reports of PBRN within primary care, we too find that studies in a PBRN setting are more resource consuming. This, in turn, means that for each involved researcher, fewer international original publications can be produced for the same cost as before. Figure 5 shows the mean number of publications of all types per full-time researcher (FTE) and a weighted score of publications where we have applied a weighted scoring system assigning original international publications two points and publications in Swedish from 1.5 to 1. As shown in the figure, the total number of publications per full-time researcher is at the same level over time. However, in line with the PBRN, there is a higher proportion of Swedish-language popular science reports, guidelines, other handouts, and types of communication targeting practitioners, which consequently lower the weighted publication score. Thus, the research may not necessarily be more expensive, but productivity as traditionally assessed by the science community will be reduced. This in turn may have negative consequences for the researcher when, for example, competing for research funding or research positions. Thus, to succeed in the PBRN aim of improving EBP, resources must be invested in an infrastructure that supports collaboration and communication in the PBRN, including costs for such aspects as communicators, a variety of means of dissemination (newsletters, time for social media, physical meetings with partners, etc.) and involvement in external popular science seminars and trainings courses. Moreover, studies in workplace settings are more demanding; as opposed to laboratory studies where you can have full control of the setting, they are generally carried out over longer periods of time, with complex evaluations rather than standardized questionnaires or only analysis of data from registers/data bases.

## 5. Conclusions

The PBRN-OSH resulted in several practice-based research projects as well as different measures to ensure EBP in OSH such as, e.g., national practice guidelines for the OH-services. Moreover, results show that the competence in EBP increased among practitioner at the OH-services. Conducting research in a PBRN is resource demanding; however, this does not imply it is less cost effective. To succeed in increasing utility of research findings via PBRN, resources must be invested in infrastructure that supports collaboration in the PBRN, including costs for a variety of means of dissemination.

### Recommendation for Improving Utilisation of Research into Practice

Researchers in the field of OSH need to be more active in disseminating their results outside the scientific community. Nationally, the importance of engaging and communicating with society is clearly emphasised by the Swedish government and research institutions. A Swedish survey among researchers shows that there is great interest in communicating and disseminating research (90%) but that few have the resources or competence to do so [32]. Researchers should therefore be rewarded for time spent on such activities. Society, universities, and funding agencies, among others, need to include this type of work in academic career paths and reward systems if a major improvement in the impact and return of investments from research is to be expected.

The final word goes to our co-authors representing the three main labour market sectors in Sweden, Private (Bodil Mellblom), Governmental (Tarja Nevala), and Local Authorities and Regions (Ned Carter): “As representative for national employer organizations, participating in this type of endeavour was well worth the time and effort involved. We support initiatives involving systematic, participatory research and development in the organizations we represent. Developing ‘best-practices’ in dialogue with those concerned seems the most promising approach to attaining methods that improve health, safety, quality, and productivity in the workplace”.

## Figures and Tables

**Table 1:** Occupational health practice guidelines developed in the program.

Occupational Health Practice Guidelines
Guideline for non-specific low back-pain (launched 2014, updated 2018) Guideline for the treatment and prevention of common mental disorders at work (launched 2015, updated 2018) Guideline for health screening at work (launched 2015, updated 2018) Guideline for alcohol problems at work (launched 2016) Guideline for measuring noise-exposure at work (launched 2016)
**Work Health Economic Analysis Tools**
Analysis tool for the treatment and prevention of common mental disorders at work (launched 2016) Analysis tool for non-specific low back-pain (launched 2018) Analysis tool for health screening at work (launched 2018)

**Table 2:** Practitioners adherence and attitudes towards the guideline for non-specific low back.

Question/Statement	*n*	Yes, % (*n*)		No, % (*n*)
Aware of the guideline	339	45.1 (153)		54.9 (186)
*Questions put to those who answered yes to the question above,* % (*n*)				
		**Yes partly**	**Yes fully**	**No**
Adhere to the guideline	142 ^#^	47.9 (68)	30.3 (43)	21.8 (31)
		**Agree**	**Partly agree**	**Disagree**
*The guideline* is clear and understandable	109	59.6 (65)	33.9 (37)	6.4 (7)
fits with our way of working	108	37 (40)	50 (54)	13.0 (14)
lay-out makes it easy to work with	105	43.8 (46)	46.7 (49)	9.5 (10)
is adaptable to our organization	108	38.0 (41)	48.1 (52)	13.9 (159)
Takes our customers preferences into consideration	106	34.0 (36)	56.6 (60)	34.0 (36)
*Barriers to implementation*				
I don’t know enough to work according to the guideline	114	12.3 (14)	27.2 (31)	60.5 (69)
Lack of managerial support	112	12.5 (14)	17.9 (20)	69.6 (78)
Guideline is difficult and complex	112	10.7 (12)	33.0 (37)	56.3 (63)
Working according to the guideline is time-consuming	110	11.8 (13)	41.8 (46)	46.4 (51)
Difficult to change routines	111	2.7 (3)	36.0 (40)	61.3 (68)

^#^*n* = number of respondents per statement excluding missing.

**Table 3:** Training, courses, and webinars aimed at capacity building for evidence-based practice (EBP) in OSH.

Courses
Course in evidence-based practice Training in evidence-based brief alcohol rehabilitation Workshop implementation of brief alcohol rehabilitation Course in alcohol and drug problems, possible interventions in practice. Course in problem-solving based dialogue method
**Seminars in Occupational Health Practice Guidelines**
Guideline for non-specific low back-pain Guideline for the treatment and prevention of common mental disorders at work Guideline for health screening at work Guideline for alcohol problems at work Guideline for measuring noise exposure at work Economic analysis tool for common mental disorders

**Table 4:** Agreement regarding involvement of researchers and partners in partnership research.

Task	Researchers	Partners
Resources (who is paying for what, intangible resources allocated, etc.)	Yes	Yes
Research design incl. intervention, method, etc.	Yes, but respect and listen to partners experiences and knowledge	Yes, but the final decisions must always be based on research quality and expertise
Data collection	Yes, responsible for designing how and when data collection should occur, material for information, etc.	Yes, if no conflict of interest, sensitive data, or other
Analysing data	Yes	No
Interpreting results	Yes, and if partners have no conflict of interest make use of the partners specific expertise of the setting/context (their workplace/work situation, etc.)	Yes, if no conflict of interest
Presenting and disseminating results	Yes	Yes

## Supplementary Materials

The following are available online at https://www.mdpi.com/1660-4601/17/15/5283/s1, Table S1: An overview of main research studies performed in the PBRN-OSH setting.

## References

[1] Hollander H., Es-Sadki N., Merkelbach I. European Innovation Scoreboard 2019. https://ec.europa.eu/growth/industry/policy/innovation/scoreboards_en.

[2] Balas E.A., Boren S.A. (2000). Yearbook of Medical Informatics.

[3] Westfall J.M., Mold J., Fagnan L. (2007). Practice-based research–“Blue Highways” on the NIH roadmap. JAMA J. Am. Med. Assoc..

[4] Green L.A., Seifert C.M. (2005). Translation of research into practice: Why we can′t “just do it”. J. Am. Board Fam. Pract..

[5] Minkler M., Salvatore A.L. (2012). Participatory Approaches for Study Design and Analysis in Dissemination and Implementation Research.

[6] Powell B.J., Waltz T.J., Chinman M.J., Damschroder L.J., Smith J.L., Matthieu M.M., Proctor E.K., Kirchner J.E. (2015). A refined compilation of implementation strategies: Results from the Expert Recommendations for Implementing Change (ERIC) project. Implement. Sci..

[7] EU-OSHA (2014). Priorities for Occupational Safety and Health Research in Europe for the Years 2013–2020.

[8] Bumbarger B.K., Campbell E.M. (2012). A state agency-university partnership for translational research and the dissemination of evidence-based prevention and intervention. Adm. Policy Ment. Health.

[9] Carpenter W.R., Meyer A.M., Wu Y., Qaqish B., Sanoff H.K., Goldberg R.M., Weiner B.J. (2012). Translating research into practice: The role of provider-based research networks in the diffusion of an evidence-based colon cancer treatment innovation. Med. Care.

[10] FORTE (2015). User Participation. Reserach with and about User Participation.

[11] Brett J., Staniszewska S., Mockford C., Herron-Marx S., Hughes J., Tysall C., Suleman R. (2014). Mapping the impact of patient and public involvement on health and social care research: A systematic review. Health Expect..

[12] Swedish Work Environment Authority The Work Environment Act. https://www.government.se/4ac754/contentassets/86e9091526644e90b78d2ff937318530/sfs-19771160-work-environment-act.

[13] Swedish Association of Occupational Health and Safety. https://www.foretagshalsor.se/.

[14] AFA Insurance. https://www.afaforsakring.se/andra-sprak/engelska/.

[15] FORTE. www.forte.se/en.

[16] Wandersman A., Duffy J., Flaspohler P., Noonan R., Lubell K., Stillman L., Blachman M., Dunville R., Saul J. (2008). Bridging the gap between prevention research and practice: The interactive systems framework for dissemination and implementation. Am. J. Commun. Psychol..

[17] Kwak L., Wahlin C., Stigmar K., Jensen I. (2017). Developing a practice guideline for the occupational health services by using a community of practice approach: A process evaluation of the development process. BMC Public Health.

[18] Jensen I., Kwak L. (2018). Intervention and Implementation Research within Occupational Health. Development and Evaluation of Cost-Effective Measures for Improved OSH. Report from the PBRN-OSH Programme.

[19] Bik H.M., Goldstein M.C. (2013). An Introduction to Social Media for Scientists. PLoS Biol..

[20] FHVforskning. http://www.fhvforskning.se/.

[21] Professornharordet. In Professornharordet. 2020, p Blog. http://www.professornharordet.se.

[22] Bramberg E.B., Nyman T., Kwak L., Alipour A., Bergstrom G., Elinder L.S., Hermansson U., Jensen I. (2017). Development of evidence-based practice in occupational health services in Sweden: A 3-year follow-up of attitudes, barriers and facilitators. Int. Arch. Occup. Environ. Health.

[23] Björk Brämberg E., Nyman T.L.K., Alipour A., Bergström G., Schäfer Elinder L., Hermansson U., Jensen I. (2015). Development of Evidence Based Practice in Occupational Health Services. A Three-Year Follow-Up of Attitudes, Barriers and Facilitators.

[24] SAWEE Myndigheten för Arbetsmiljökunskap. https://www.mynak.se/.

[25] Brownson R.C., Baker E.A., Deshpande A.D., Gillespie K.N. (2018). Evidence-Based Public Health.

[26] Barreto J.E., Whitehair C.L. (2017). Social Media and Web Presence for Patients and Professionals: Evolving Trends and Implications for Practice. PM&R.

[27] Brown C.H., Curran G., Palinkas L.A., Aarons G.A., Wells K.B., Jones L., Collins L.M., Duan N., Mittman B.S., Wallace A. (2017). An Overview of Research and Evaluation Designs for Dissemination and Implementation. Annu. Rev. Public Health.

[28] Gordan V.V., Makhija S.K., Rindal D.B., Meyerowitz C., Fellows J.L., Ziegenfuss J.Y., Cochran D.L., Hudak S., Gilbert G.H., National Dental PBRN Collaborative Group (2019). Leadership in practice-based research: The National Dental PBRN. J. Dent..

[29] Fisher M., Brewer S.E., Westfall J.M., Simpson M., Zittleman L., O’Leary S.T., Fernald D.H., Nederveld A., Nease D.E. (2019). Strategies for Developing and Sustaining Patient and Community Advisory Groups: Lessons from the State Networks of Colorado Ambulatory Practices and Partners (SNOCAP) Consortium of Practice-Based Research Networks. J. Am. Board Fam. Med..

[30] Durlak J.A., DuPre E.P. (2008). Implementation matters: A review of research on the influence of implementation on program outcomes and the factors affecting implementation. Am. J. Commun. Psychol..

[31] Westfall J.M., Roper R., Gaglioti A., Nease D.E. (2019). Practice-Based Research Networks: Strategic Opportunities to Advance Implementation Research for Health Equity. Ethn. Dis..

[32] Bohlin G., Bergman M. (2019). Researchers’ Views on Communication and Open Science in Sweden.

