# Supplementary material for: Promoting Evidence-Based Practice for Improved Occupational Safety and Health at Workplaces in Sweden. Report on a Practice-Based Research Network Approach
Source: Int J Environ Res Public Health. 2020 Jul 22;17(15):5283. doi: 10.3390/ijerph17155283 (PMC7432656; doi:10.3390/ijerph17155283)
Supplement: Supplementary file 1 [file ijerph-17-05283-s001.pdf]

Appendix 1. An overview of main research studies performed in the PBRN-OSH setting.

	**Design**	**Involved partners**	**International Publications**
The Stress Prevention At Work (SPA)-study	RCT, Mixed methods	Primary Health care units and OH service at one county council	[1-3]
Problem solving intervention for CMD at work (PIA)-study	RCT, Mixed methods<br>Cost effectiveness	OH-services on different locations in Sweden	[4,5]
Prevention of common mental disorders at the workplace" in schools The iSkol-study	RCT, Mixed methods<br>Implementation study design	Three municipalities in Sweden	[6,7]
Bullying in academia and industry	Cohort study<br>Mixed methods	Labour union for managers (Ledarna, Unionen)	[8,9]
Effectiveness of yoga for employees with back pain (RYS)-study	RCT<br>Cost effectiveness	OH-services, fitness center, Yoga center	[10,11]
Measuring production loss due to ill health and work-environment problems.	Validation studies and cohort study, survey	Local work places	[12-16]
What incentives influence employers to engage in workplace health interventions	Cohort study, Interviews	Local medium to large companies	[17]
Preferences for physical activity when suffering from LBP	Discrete choice design	OH-services on different locations	[18,19]
Promoting EBP in OSH	Cohort study, Survey, interviews	The Swedish association for occupational health	[20,21]
Work environment and research productivity	Cohort study, Survey and register	A medical university in Sweden	[22]
Economic Evaluation of Occupational Safety and Health Interventions From the Employer Perspective	Systematic review		[23]
Interventions for common mental disorders in the occupational health service	Systematic review		[24]
The effectiveness of workplace nutrition and physical activity interventions in improving productivity, work performance and workability.	Systematic review		[25]

1. Arapovic-Johansson, B.; Wåhlin, C.; Hagberg, J.; Kwak, L.; Björklund, C.; Jensen, I. Participatory work place intervention for stress prevention in primary health care. A randomized controlled trial. *Eur J Work Organ Psy* **2018**, 10.1080/1359432X.2018.1431883, 1-16, doi:10.1080/1359432X.2018.1431883.
2. Arapovic-Johansson, B.; Wahlin, C.; Hagberg, J.; Kwak, L.; Axen, I.; Bjorklund, C.; Jensen, I. Experience of Stress Assessed by Text Messages and Its Association with Objective Workload-A Longitudinal Study. *Int J Env Res Pub He* **2020**, *17*, doi:ARTN 680 10.3390/ijerph17030680.
3. Arapovic-Johansson, B.; Wahlin, C.; Kwak, L.; Bjorklund, C.; Jensen, I. Work-related stress assessed by a text message single-item stress question. *Occup Med-Oxford* **2017**, *67*, 601-608, doi:10.1093/occmed/kqx111.

4. Bergstrom, G.; Lohela-Karlsson, M.; Kwak, L.; Bodin, L.; Jensen, I.; Torgen, M.; Nybergh, L. Preventing sickness absenteeism among employees with common mental disorders or stress-related symptoms at work: Design of a cluster randomized controlled trial of a problem-solving based intervention versus care-as-usual conducted at the Occupational Health Services. *Bmc Public Health* **2017**, *17*, doi:ARTN 436 10.1186/s12889-017-4329-1.

5. Keus van de Poll, M.; Nybergh, L.; Lornudd, C.; Hagberg, J.; Bodin, L.; Kwak, L.; Jensen, I.; Lohela-Karlsson, M.; Torgén, M.; Bergström, G. Preventing sickness absence among employees with common mental disorders or stress-related symptoms at work: a cluster randomised controlled trial of a problem-solving-based intervention conducted by the Occupational Health Services. *Occup Environ Med* **2020**, *0*, 1-8, doi:http://dx.doi.org/10.1136/oemed-2019-106353.
6. Kwak, L.; Lornudd, C.; Bjorklund, C.; Bergstrom, G.; Nybergh, L.; Elinder, L.S.; Stigmar, K.; Wahlin, C.; Jensen, I. Implementation of the Swedish Guideline for Prevention of Mental ill-health at the Workplace: study protocol of a cluster randomized controlled trial, using multifaceted implementation strategies in schools. *Bmc Public Health* **2019**, *19*, doi:ARTN 1668 10.1186/s12889-019-7976-6.

7. Bostrom, M.; Bjorklund, C.; Bergstrom, G.; Nybergh, L.; Elinder, L.S.; Stigmar, K.; Wahlin, C.; Jensen, I.; Kwak, L. Health and Work Environment among Female and Male Swedish Elementary School Teachers-A Cross-Sectional Study. *Int J Env Res Pub He* **2020**, *17*, doi:ARTN 227 10.3390/ijerph17010227.

8. Bjorklund, C.; Hellman, T.; Jensen, I.; Akerblom, C.; Bramberg, E.B. Workplace Bullying as Experienced by Managers and How They Cope: A Qualitative Study of Swedish Managers. *Int J Env Res Pub He* **2019**, *16*, doi:ARTN 4693 10.3390/ijerph16234693.

9. Bjorklund, C.; Vaez, M.; Jensen, I. Early work-environmental indicators of bullying in an academic setting: a longitudinal study of staff in a medical university. *Stud High Educ* **2020**, 10.1080/03075079.2020.1729114, doi:10.1080/03075079.2020.1729114.
10. Aboagye, E.; Karlsson, M.L.; Hagberg, J.; Jensen, I. Cost-effectiveness of early interventions for non-specific low back pain: a randomized controlled study investigating medical yoga, exercise therapy and self-care advice. *J Rehabil Med* **2015**, *47*, 167-173, oi:10.2340/16501977-1910.
11. Bramberg, E.B.; Bergstrom, G.; Jensen, I.; Hagberg, J.; Kwak, L. Effects of yoga, strength training and advice on back pain: a randomized controlled trial. *Bmc Musculoskel Dis* **2017**, *18*, doi:ARTN 132 10.1186/s12891-017-1497-1.

12. Lohela Karlsson, M.; Busch, H.; Aboagye, E.; Jensen, I. Validation of a measure of health-related production loss: construct validity and responsiveness - a cohort study. *BMC Public Health* **2015**, *15*, 1148, doi:10.1186/s12889-015-2449-z.
13. Aboagye, E.; Jensen, I.; Bergstrom, G.; Hagberg, J.; Axen, I.; Lohela-Karlsson, M. Validity and test-retest reliability of an at-work production loss instrument. *Occup Med (Lond)* **2016**, 10.1093/occmed/kqw021, doi:10.1093/occmed/kqw021.
14. Stromberg, C.; Aboagye, E.; Hagberg, J.; Bergstrom, G.; Lohela-Karlsson, M. Estimating the Effect and Economic Impact of Absenteeism, Presenteeism, and Work Environment-Related Problems on Reductions in Productivity from a Managerial Perspective. *Value Health* **2017**, *20*, 1058-1064, doi:10.1016/j.jval.2017.05.008.
15. Karlsson, M.L.; Bergstrom, G.; Bjorklund, C.; Hagberg, J.; Jensen, I. Measuring Production Loss due to Health and Work Environment Problems Construct Validity and Implications. *Journal of Occupational and Environmental Medicine* **2013**, *55*, 1475-1483, doi:10.1097/Jom.0000000000000005.

16. Karlsson, M.L.; Busch, H.; Aboagye, E.; Jensen, I. Validation of a measure of health-related production loss: construct validity and responsiveness - a cohort study. *Bmc Public Health* **2015**, *15*, doi:ARTN 1148 10.1186/s12889-015-2449-z.

17. Martinsson, C.; Lohela-Karlsson, M.; Kwak, L.; Bergstrom, G.; Hellman, T. What incentives influence employers to engage in workplace health interventions? *Bmc Public Health* **2016**, *16*, doi:ARTN 854 10.1186/s12889-016-3534-7.

18. Aboagye, E.; Hagberg, J.; Axen, I.; Kwak, L.; Lohela-Karlsson, M.; Skillgate, E.; Dahlgren, G.; Jensen, I. Individual preferences for physical exercise as secondary prevention for non-specific low back pain: A discrete choice experiment. *Plos One* **2017**, *12*, doi:ARTN e0187709 10.1371/journal.pone.0187709.

19. Aboagye, E. Valuing Individuals' Preferences and Health Choices of Physical Exercise. *Pain Ther* **2017**, *6*, 85-91, doi:10.1007/s40122-017-0067-4.

20. Bramberg, E.B.; Nyman, T.; Kwak, L.; Alipour, A.; Bergstrom, G.; Elinder, L.S.; Hermansson, U.; Jensen, I. Development of evidence-based practice in occupational health services in Sweden: a 3-year follow-up of attitudes, barriers and facilitators. *Int Arch Occ Env Hea* **2017**, *90*, 335-348, doi:10.1007/s00420-017-1200-8.

21. Kwak, L.; Wahlin, C.; Stigmar, K.; Jensen, I. Developing a practice guideline for the occupational health services by using a community of practice approach: a process evaluation of the development process. *BMC Public Health* **2017**, *17*, 89, doi:10.1186/s12889-016-4010-0.

22. Jensen, I.; Bjorklund, C.; Hagberg, J.; Aboagye, E.; Bodin, L. An overlooked key to excellence in research: a longitudinal cohort study on the association between the psycho-social work environment and research performance. *Stud High Educ* **2020**, 10.1080/03075079.2020.1744127, doi:10.1080/03075079.2020.1744127.

23. Grimani, A.; Bergstrom, G.; Casallas, M.I.R.; Aboagye, E.; Jensen, I.; Lohela-Karlsson, M. Economic Evaluation of Occupational Safety and Health Interventions From the Employer Perspective A Systematic Review. *Journal of Occupational and Environmental Medicine* **2018**, *60*, 147-166, doi:10.1097/Jom.0000000000001224.

24. Axen, I.; Bramberg, E.B.; Vaez, M.; Lundin, A.; Bergstrom, G. Interventions for common mental disorders in the occupational health service: a systematic review with a narrative synthesis. *Int Arch Occ Env Hea* **2020**, 10.1007/s00420-020-01535-4, doi:10.1007/s00420-020-01535-4.

25. Grimani, A.; Aboagye, E.; Kwak, L. The effectiveness of workplace nutrition and physical activity interventions in improving productivity, work performance and workability: a systematic review. *Bmc Public Health* **2019**, *19*, doi:ARTN 1676 10.1186/s12889-019-8033-1.
